# Effects of Fermented Ginseng By-Product Supplementation on Growth Performance, Meat Quality, Flavor, and Fatty Acid Metabolism in Hongyu Roosters

**DOI:** 10.3390/vetsci13080844

**Published:** 2026-08-21

**Authors:** Hongxi Chen, Yuhao Liu, Shuo Zhang, Yi Jin, Wanfeng Liang, Xijiu Jin, Junzhe Min

**Affiliations:** 1 Key Laboratory of Natural Medicines of the Changbai Mountain, Ministry of Education, Department of Phamaceutical Analysis, College of Pharmacy, Yanbian University, Yanji 133002, China; 2 Department of Animal Science, College of Agriculture, Yanbian University, Yanji 133002, China

**Keywords:** Hongyu roosters, fermented ginseng by-product, fermented feed, growth performance, meat quality, untargeted metabolomics

## Abstract

Agricultural by-products and Chinese medicine crop residues, as non-traditional animal feed, have great potential to alleviate the scarcity of traditional feed and reduce the environmental burden. In this study, the effects of adding fermented ginseng by-products to the diet on growth performance, meat quality, flavor characteristics and metabolism of Hongyu roosters were evaluated. A total of 100 Hongyu roosters were randomly assigned to either a control (Con) group fed a basal diet or a fermented ginseng by-products (Fe) group fed a basal diet supplemented with 3% fermented ginseng by-products, with five replicates per group, for 100 days. The results demonstrated that the fermented diet significantly enhanced final body weight and average daily gain (ADG) while reducing the feed conversion ratio (FCR) (*p* < 0.05). The percentages of breast and thigh muscles and breast muscle fiber diameter were significantly increased. The results also exhibited enhanced water-holding capacity, coupled with significantly decreased shear force and drip loss (*p* < 0.05). Electronic nose and tongue analyses confirmed enriched meat aroma and optimized taste profiles, which aligned with elevated levels of flavor-precursor amino acids and essential polyunsaturated fatty acids. Serum metabolomics found purine metabolism and the biosynthesis of unsaturated fatty acids to be pivotal pathways. Metabolites related to nucleotide and lipid metabolism were regulated. In conclusion, fermented ginseng by-products serve as an effective functional feed to improve poultry growth performance and meat quality.

## Simple Summary

This study explores the use of fermented ginseng by-products as a non-traditional feed alternative to address traditional feed scarcity and agricultural waste. We evaluated the effects of adding these fermented residues to the diet of Hongyu roosters, focusing on growth performance, meat quality, flavor characteristics, and metabolic changes. The results demonstrate that the fermented diet significantly boosts growth performance, muscle development, and meat tenderness while enhancing desirable flavor profiles and beneficial polyunsaturated fatty acids. Metabolomics further reveals that improvements are driven by optimized purine metabolism and unsaturated fatty acid biosynthesis. Ultimately, this research shows that fermented ginseng by-products serve as an effective functional feed, offering a sustainable strategy to elevate poultry production quality and reduce environmental burdens.

## 1. Introduction

The continuous growth of global crop yield leads to a large number of crop residues. China generates approximately 900 million tons of crop residues annually, representing a substantial renewable biomass resource that requires efficient utilization [1]. However, despite the availability of various utilization approaches, including animal feeding, bioenergy production, composting, and industrial applications, a considerable proportion of crop residues remains underutilized or improperly managed, causing environmental concerns and limiting their potential value [2]. Therefore, the effective utilization of crop residues is very important for environmental health and sustainable development of agriculture. Crop residues are mainly used as animal feed and organic fertilizer [3]. Feed represents the largest expense in poultry production, generally accounting for approximately 75% of total production costs [4]. The increasing demand for grains for food consumption and biofuel production has contributed to substantial increases in the prices of major feed ingredients, particularly corn and soybean meal. These challenges have intensified the economic pressure on poultry producers and highlighted the urgent need to develop sustainable and cost-effective alternative feed resources [5]. Therefore, the use of agricultural and sideline products and crop residues as economical and easily available substitutes for traditional feed raw materials have attracted attention [5]. At present, the demand for livestock and poultry products in most developing countries continues to rise, and many countries still face the problem of feed shortages [6]. Therefore, the utilization of agro-industrial by-products, fermented herbal feed additives, and fermented medicinal plant by-products as alternative feed resources has attracted increasing attention. Among these alternatives, medicinal plant residues have gained particular interest. Ginseng residue was a suitable feed additive for improving production performance and health in sika deer [7]. The combined supplementation of ginseng polysaccharides with fermented feed has been reported to further improve growth performance and immune parameters in Xuefeng black-bone chickens, while reducing diarrhea incidence and mortality [8]. Dandelion has great potential to be used as a feed additive. Supplementation with 500 mg/kg of fermented dandelion reduced the L* and b* values and shear force of breast muscle, thereby improving the performance and health status of broilers [9]. Overall, it is very important to use non-traditional feed sources such as agricultural and sideline products and crop residues to build a sustainable poultry feeding scheme.

In order to overcome the current challenges in feed resource development and improve animal health performance, microbial fermentation technology can be applied to improve the nutritional and functional value of unconventional feed resources [10,11]. Microbial fermentation is a low-cost and promising feed processing technology, which can enhance the nutritional value and functional characteristics of feed and improve its bioavailability [12,13]. Previous studies have indicated that fermented feed has the potential to enhance growth performance, antioxidant capacity, immune responses, and metabolic profiles in poultry; however, these effects may vary depending on the fermentation substrate, microbial strains, processing conditions, and dietary inclusion levels [14,15]. Fermentation is widely used in the animal feed industry. The fermentation of agricultural and industrial by-products can increase their protein content and reduce their fiber content [16]. Various microorganisms are commonly used in feed fermentation, with *Bacillus* spp., *Lactobacillus* spp., and *Saccharomyces* spp. being among the most widely applied strains. *Bacillus* spp. possess strong extracellular enzyme-producing capabilities and can secrete proteases, cellulases, amylases, and phytases, thereby facilitating the degradation of complex plant components and improving nutrient availability [17]. *Lactobacillus* spp. produce lactic acid and other organic acids, which contribute to pH reduction, inhibition of harmful microorganisms, and modulation of intestinal microbial communities [18]. In contrast, *Saccharomyces* spp. can generate various beneficial metabolites, including vitamins, amino acids, yeast polysaccharides, and other bioactive compounds, which may enhance feed nutritional value and regulate animal immune responses [19]. Microbial fermentation improves feed quality through multiple biological processes. During fermentation, microorganisms produce various extracellular enzymes that can degrade complex plant cell wall components, reduce fiber content, and enhance nutrient accessibility and digestibility [20,21]. In addition, fermentation can decrease anti-nutritional factors, promote the release of soluble nutrients, and generate microbial-derived proteins and small bioactive peptides, thereby improving the nutritional value of feed resources [22,23]. For ginseng-derived materials, microbial fermentation can facilitate the biotransformation of major ginsenosides into rare ginsenosides with higher bioavailability and biological activity [24,25]. Moreover, fermented feed has been reported to modulate intestinal microbiota composition and metabolism, contributing to improved nutrient utilization and overall health status in animals [5].

*Panax ginseng*, a well-known medicinal plant, contains various bioactive components, including ginsenosides, polysaccharides, amino acids, peptides, and phenolic compounds. Among these constituents, ginsenosides are considered the major functional compounds responsible for its antioxidant, anti-inflammatory, and immunomodulatory activities. In animal nutrition, ginseng-derived products have attracted increasing attention due to their potential to enhance antioxidant status, regulate immune responses, improve intestinal health, and promote growth performance in poultry. Studies have demonstrated that dietary supplementation with ginseng polysaccharides or ginseng-derived products could improve production performance, intestinal development and immune parameters in poultry, suggesting their potential application as functional feed additives [26,27]. A large number of ginseng by-products such as powder and fiber roots are produced during the processing of ginseng [28]. Although most water-soluble active compounds are removed during processing, ginseng residue still contains crude protein, fiber components, residual ginsenosides, polysaccharides, and mineral components [29]. In poultry, ginseng by-products have been proven to increase the egg production of 75-week-old Hy-Line brown chickens [30] and reduce the mortality of white feather broilers [31]. The direct application of whole ginseng or ginseng extracts in animal feed is limited due to their high economic value and processing costs. In contrast, ginseng by-products generated during industrial processing are abundant, underutilized resources that retain considerable bioactive components and have potential value for further utilization. However, their application is restricted by poor digestibility and bioavailability. Fermentation technology provides an effective approach to improve the nutritional characteristics and functional properties of ginseng by-products [32], making them promising alternatives for sustainable poultry production. Under modern intensive production systems, livestock and poultry are often exposed to various environmental and management-related stressors, such as heat stress, oxidative stress, transportation stress, and pathogen challenges, which may impair immune function and reduce production efficiency. Therefore, the development of functional feed additives with antioxidant and immunomodulatory properties has become an important strategy for improving animal health and performance. Due to its diverse bioactive compounds, including ginsenosides and polysaccharides, ginseng-derived products have attracted increasing attention as potential functional feed resources in animal production.

Hongyu roosters are a commercial crossbred meat-type chicken developed through the hybridization of Anka red broilers and Babcock B380 chickens. This breed is characterized by a relatively large body size, small head, distinctive red plumage, and good disease resistance [33]. To date, the effects of fermented ginseng by-products as a feed additive in Hongyu roosters have not been investigated. Based on these considerations, we hypothesized that dietary supplementation with fermented ginseng by-products could improve growth performance, meat quality, and flavor characteristics in Hongyu roosters by enhancing nutrient availability and bioactive compound utilization. Therefore, this study aimed to establish a scientific basis for the application of fermented ginseng by-products as a functional feed ingredient by investigating their effects on growth performance, meat quality, and metabolic regulation, thereby providing new insights into the sustainable utilization of ginseng processing residues in poultry production.

## 2. Materials and Methods

### 2.1. Animal Ethics

This research adhered to animal experimentation ethics standards and received approval from the Animal Ethics Committee of Yanbian University for the protection of animals used in scientific research (approval No. YD20240827005).

### 2.2. Preparation of Fermented Feed

The ginseng by-products were obtained from Han Zheng Ginseng Factory in Yanji, China. These by-products consisted of residual ginseng material remaining after the extraction of active components. Ginseng fermentation inoculum is a solid powder fermented by five kinds of microorganisms, including *Lactobacillus plantarum*, *Lactobacillus cellobiosus*, *Lactobacillus brevis*, *Bacillus lentus* and *Rhodopseudomonas palustris* in a 1:1:1:1:1 proportion. Ginseng by-products were inoculated with the composite microbial inoculum at 0.1% (*w/w*). Corn flour ground to approximately 40 mesh (particle size approximately 0.42 mm) was used as an auxiliary solid fermentation substrate. After sterilization, the inoculated substrate was subjected to solid-state fermentation in polypropylene fermentation trays at 30 °C for 48 h without additional aeration. The moisture content of the fermentation substrate was adjusted to 60%. After fermentation, the products were vacuum-dried at 30 °C and ground into powder. The viable microbial count was determined using the plate colony counting method, and the final fermented product maintained a viable microbial count of ≥2.0 × 10^8^ CFU/g, with microbial survival rates above 90% after low-temperature drying.

### 2.3. Feed Metabolomics Analysis Sample Preparation

A total of 200 mg (*n* = 9) of feed sample was accurately weighed and extracted with 1 mL methanol via sonication for 30 min. The mixture was centrifuged at 7378× *g* for 10 min, and the supernatant was dried at 30 °C. The residue was reconstituted in 1 mL of 75% methanol and centrifuged again at 7378× *g*. The resulting supernatant was filtered through a 0.22 μm millipore filter for UHPLC-HRMS analysis.

### 2.4. Animals and Experimental Design

**Figure 1:**
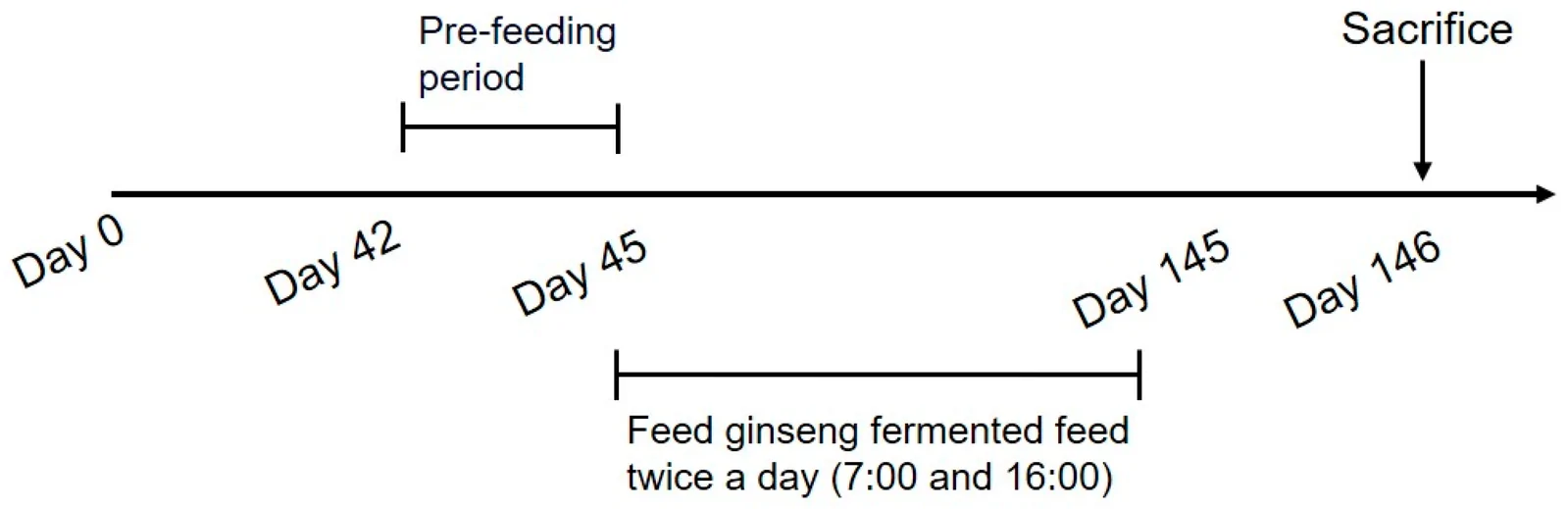
Experimental flow chart.

**Figure 2:**
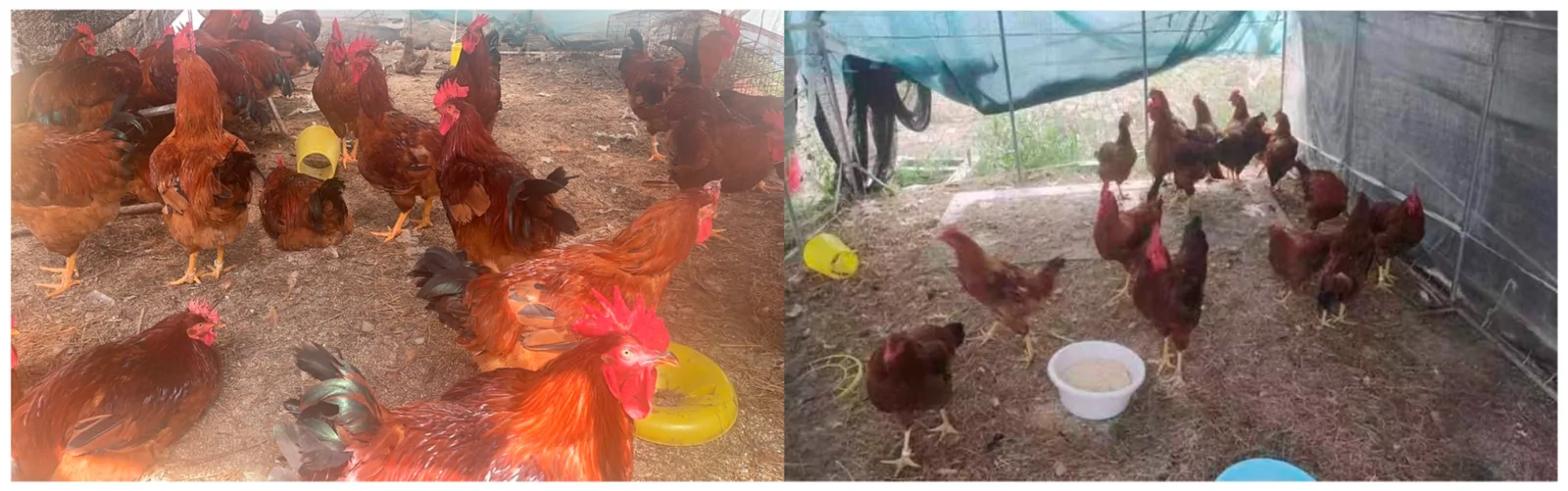
Two identical henhouses.

A completely randomized design was used in this experiment. After a 3-day adaptation period and weighing all experimental Hongyu roosters, 100 healthy Hongyu roosters (45 days old; initial body weight: 0.69 ± 0.16 kg) with similar initial body weights were selected and individually identified using leg bands. The selected roosters were randomly allocated to the control group (Con) and fermented ginseng by-product group (Fe). Subsequently, Hongyu roosters within each treatment were randomly assigned to five replicates, with 10 birds per replicate. The experimental procedure is shown in Figure 1. Hongyu roosters within each replicate were housed together, and each replicate was considered an independent experimental unit. The experiment was conducted in an open breeding area located behind the Animal Hospital of the Agricultural College of Yanbian University. Two identical henhouses were constructed to simulate the free-range farming conditions commonly used in local poultry production (Figure 2). Feeding occurred twice daily (07:00 and 16:00), with ad libitum access to food and water. Before the experiment commenced, the henhouses were thoroughly cleaned and disinfected. Throughout the study, conditions were maintained to ensure cleanliness, dryness, and proper hygiene of the henhouses and feeding troughs, with consistent lighting and ventilation.

### 2.5. Diet Composition and Nutritional Level Analysis

To meet the nutritional requirements of Hongyu roosters, the experimental diet was formulated in accordance with the Nutritional Requirements of Yellow Feather Roosters (NY/T 3645-2020) [34]. Nutrient composition of the experimental diet samples was analyzed according to the recommended national standard of China (GB/T): crude protein (GB/T 6432-2018 [35], Kjeldahl method, N × 6.25), calcium and phosphorus (GB/T 6436-2018 [36], spectrophotometry after acid digestion), amino acids (GB/T 18246-2019 [37], high-performance liquid chromatography). The metabolizable energy (ME) was calculated according to GB/T 26437-2010 [38], Determination of Metabolizable Energy in Feed; the specific composition is shown in Table 1.

### 2.6. Sample Collection

At the end of the feeding trial, two Hongyu roosters were randomly selected from each replicate, yielding a total of 10 roosters per group across five replicates. After being fasted for 12 h, all selected roosters were subjected to slaughter, and muscle tissues were harvested. Blood samples were drawn from the brachial vein and centrifuged at 664× *g* for 15 min. The serum supernatant was collected and stored at −80 °C for the analysis of biochemical parameters and untargeted metabolomics. Following electrical stunning, the experimental roosters were slaughtered via carotid artery exsanguination for tissue samples. Internal organs were excised and weighed to calculate organ indices. Breast muscle samples were fixed in 4% paraformaldehyde at room temperature for 24–48 h for subsequent histological evaluation. Furthermore, an electronic nose and electronic tongue system were employed to analyze the sensory attributes of the meat samples.

### 2.7. Growth Performance Calculation

Roosters were weighed individually at the beginning (45 days of age) and end (145 days of age) of the experimental period after a 12 h fasting period. Initial and final body weights were recorded to calculate the average daily gain. Additionally, daily feed intake was monitored to determine the average daily feed intake and feed conversion ratio. Growth performance parameters were calculated as follows:

Average daily feed intake (ADFI) = Total feed intake/(Number of roosters × Experimental days)

Feed conversion ratio (FCR) = Feed intake/Live body weight gain

### 2.8. Measurement of Carcass Performance

The live body weight was measured and recorded prior to slaughter. After slaughter and wet pulling, the epidermis, toenails and beak sheath of the foot were removed to obtain dressed weight. Subsequently, the reproductive organs, intestines, esophagus, trachea, tumor, pancreas, spleen, gallbladder, gizzard contents and epidermis were removed to determine the semi-eviscerated weight. For eviscerated weight, the roe, anterior chamber, liver, heart, lung, abdominal fat, head and feet were removed, and the abdominal fat was collected and weighed separately. The chest and thigh muscles were dissected from both sides of the carcass and weighed separately. Methods referred to “Terms and Measurement Statistical Methods of Poultry Production Performance (NY/T 823-2020) [39].”

### 2.9. Measurement of Conventional Meat Quality

Samples of breast muscle were collected immediately after slaughter for meat quality evaluation. Muscle pH was determined 45 min post-mortem (pH_45min_). After 24 h (pH_24h_), the pH value was re-evaluated (4 °C). The color of breast muscle was measured after chilling for 24 h. The measurements of lightness (L*), redness (a*), and yellowness (b*) were performed on the same anatomical region of each sample. Specifically, we selected three fixed spots from the central area of the cross-sectional surface of the pectoralis major muscle, avoiding visible fat, connective tissue, and blood spots, and used the average value for statistical analysis. The sample was suspended in a sealed bag at 4 °C for 24 h and reweighed to calculate the drip loss. Each cooked sample was sliced parallel to the direction of muscle fibers, and the shear force perpendicular to the fiber direction was measured with a tenderness meter.

### 2.10. HE Staining of Chest Muscle Fibers

Muscle tissue samples were prepared as strips measuring 1 cm × 1 cm × 2 cm, fixed in 4% paraformaldehyde for 48 h, and then rinsed under running water for 2 h. After paraffin embedding and sectioning, HE staining was used to observe the chest muscle fibers. Muscle fiber slices were imaged with an OLYMPUS fluorescence microscope at 10 × 40 magnification, and muscle fiber density and diameter were quantified using ImageJ software (version 1.54d). The muscle fiber diameter was calculated by averaging the long and short axes of each fiber, while muscle fiber density was determined by counting the number of fibers per unit area and converting to the mean value of fibers per mm^2^.

### 2.11. Organ Index

The thymus, spleen, bursa of Fabricius, liver, kidneys, heart, lungs, proventriculus, gizzard, and pancreas were excised and weighed individually. The organ index was calculated using the formula:

organ index = organ weight (g)/live body weight (g) × 1000

### 2.12. Electronic Nose Analysis of Chest Muscle

For the electronic nose analysis, 5 g of chicken meat sample was minced and transferred to a 20 mL headspace vial at room temperature. Following a 15 min gas equilibration, the volatile substances were analyzed by inserting the electronic nose probe into the headspace. The measurement conditions were configured with a sampling interval of 1 s, flushing for 200 s, zeroing for 10 s, sample preparation for 5 s, and analysis for 100 s, under a carrier gas flow rate and sample injection flow rate of 200 mL/min. The sensor signals stabilized after 95 s, and the data acquisition window was selected at 97–99 s. Results were analyzed using Win Muster software (version 1.6.2).

### 2.13. Electronic Tongue Analysis of Chest Muscle

For electronic tongue analysis, 9 g of minced chicken meat was homogenized with 90 mL of distilled water (1:10, *w/v*) and centrifuged at 7378× *g* for 10 min. The mixture was then filtered through three layers of gauze, and the collected filtrate was placed into an E-tongue-specific beaker for further analysis. The Sample_measurement (2Stes_washing) program was used to evaluate five basic tastes: sourness, bitterness, astringency, umami, and saltiness.

### 2.14. Determination of Blood Biochemical Indices

Blood biochemical indices were determined using a WASSON Charm 2000 automatic biochemical analyzer (Wasson Medical, Beijing, China), including red blood cell, hemoglobin, hematocrit, erythrocyte sedimentation, blood platelet, white blood cell, neutrophil, eosinophilic granulocyte, basophils, lymphocyte, monocyte, total protein, albumin, α-globulin, β-globulin, γ-globulin, Na, K, Cl, inorganic phosphorus, Ca, Mg, glucose and urea nitrogen levels.

### 2.15. Serum Non-Targeted Metabonomics Sample Preparation

For serum metabolomics analysis, one Hongyu rooster was randomly selected from each replicate of the two treatment groups, resulting in five biological samples per group (*n* = 5/group; 10 samples in total). Each selected bird represented one independent biological replicate corresponding to one experimental replicate. Plasma samples (100 μL, *n* = 10) from each group were treated with 400 μL of precooled methanol to precipitate proteins using vortex mixing. The samples were then centrifuged at 7378× *g* for 10 min, and the supernatant was evaporated at 35 °C. A total of 200 μL of a 75% methanol solution was added for redissolution followed by centrifugation at 7378× *g*. The supernatant was filtered through a 0.22 μm millipore filter for untargeted metabolomics analysis of broiler plasma differential metabolites.

### 2.16. Metabolomics Analysis

Untargeted metabolomic analyses of feed and serum samples were performed using ultra-high-performance liquid chromatography coupled with high-resolution mass spectrometry (UHPLC-HRMS). Chromatographic separation was achieved using a Vanquish Flex UHPLC system (Thermo Fisher Scientific, Waltham, MA, USA) equipped with a Waters ACQUITY BEH C18 column (100 × 2.1 mm, 1.7 μm). The mobile phases consisted of 0.1% formic acid in water (A) and 0.1% formic acid in methanol (B), with a flow rate of 0.35 mL/min and an injection volume of 5 μL. The column temperature was maintained at 40 °C. The gradient elution profile was set as 0–40 min, 5–100% (B). Mass spectrometric data were acquired using a Q-Exactive Orbitrap mass spectrometer (Thermo Fisher Scientific, Waltham, MA, USA) operating in both positive and negative electrospray ionization modes. Full MS scans were acquired over an *m*/*z* range of 80–1200 with a resolution of 70,000, while MS/MS spectra were collected using the data-dependent acquisition mode with a resolution of 17,000 and stepped normalized collision energies of 20, 40, and 60 eV. Quality control (QC) samples were prepared by pooling equal volumes of all individual samples and were injected after every six experimental samples throughout the analytical sequence to monitor instrument stability and analytical reproducibility. Raw data were processed using Compound Discoverer 3.2 software (Thermo Fisher Scientific, Waltham, MA, USA). Following automatic peak extraction, alignment, and peak convolution, background ions and metabolic features with a coefficient of variation (CV) > 30% in QC samples were excluded to improve data quality and analytical reproducibility. The remaining features were subsequently subjected to multivariate statistical analyses, including PCA, volcano plot analysis, and HCA. Metabolite identification was performed by matching accurate masses, isotope patterns, and MS/MS fragmentation information against HMDB (https://www.hmdb.ca/) and ChemSpider (https://www.chemspider.com/). Pathway enrichment was performed using the KEGG database on MetaboAnalyst 6.0 (https://dev.metaboanalyst.ca/MetaboAnalyst/ (accessed on 17 August 2026)). OPLS-DA models were constructed using SIMCA-P (version 14.1, Umetrics AB, Umeå, Sweden) to visualize metabolic differences between groups. The robustness of the models was evaluated by 200 permutation tests, and R^2^Y and Q^2^ values were used to assess model fitting and predictive performance. Differential metabolites were identified based on fold change (FC > 3) and statistical significance (*p* < 0.05 after FDR correction).

### 2.17. Amino Acids and Fatty Acid Analysis

The determination of fatty acids and amino acids in meat samples was entrusted to Jilin Academy of Agricultural Sciences for analysis. Fatty acid analysis was conducted via gas chromatography following the national standard GB 5009.168-2016 [40] “Determination of Fatty Acids in Meat and Meat Products.” Amino acid composition and content in chicken meat were determined with an automatic amino acid analyzer (A300, Dalian Elite Analytical Instruments Co., Ltd., Dalian, China) according to the national standard GB 5009.124-2016 [41], “Determination of Amino Acids in Foods.”

### 2.18. Statistical Analysis

Statistical analyses were performed using SPSS software (version 17.0; IBM Corp., Armonk, NY, USA). The replicate pen was considered the experimental unit. For growth performance parameters, each replicate pen was treated as one independent observation, resulting in five experimental units per dietary treatment. Two roosters with body weights close to the average body weight of each pen were randomly selected from each pen. The measurements obtained from these two roosters were averaged to generate one pen-level observation. Differences between the Con and Fe groups were analyzed using an independent Student’s *t*-test. A value of *p* < 0.05 was considered statistically significant.

## 3. Results

### 3.1. Differential Chemical Constituents in Fermented Ginseng Feed and Basal Feed

**Figure 3:**
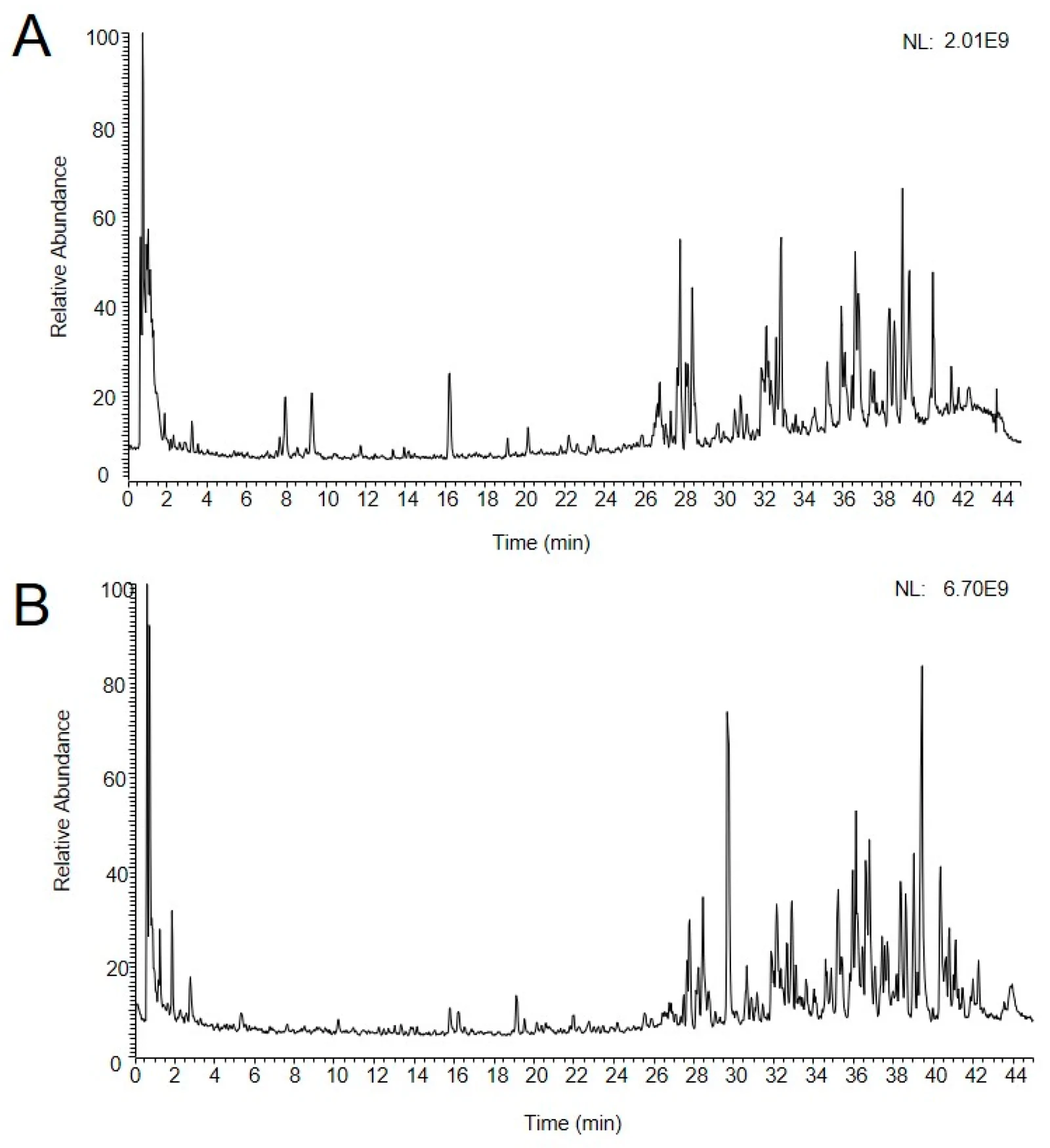
Total ion chromatogram of fermented ginseng feed. (**A**) Negative ion mode. (**B**) Positive ion mode.

**Figure 4:**
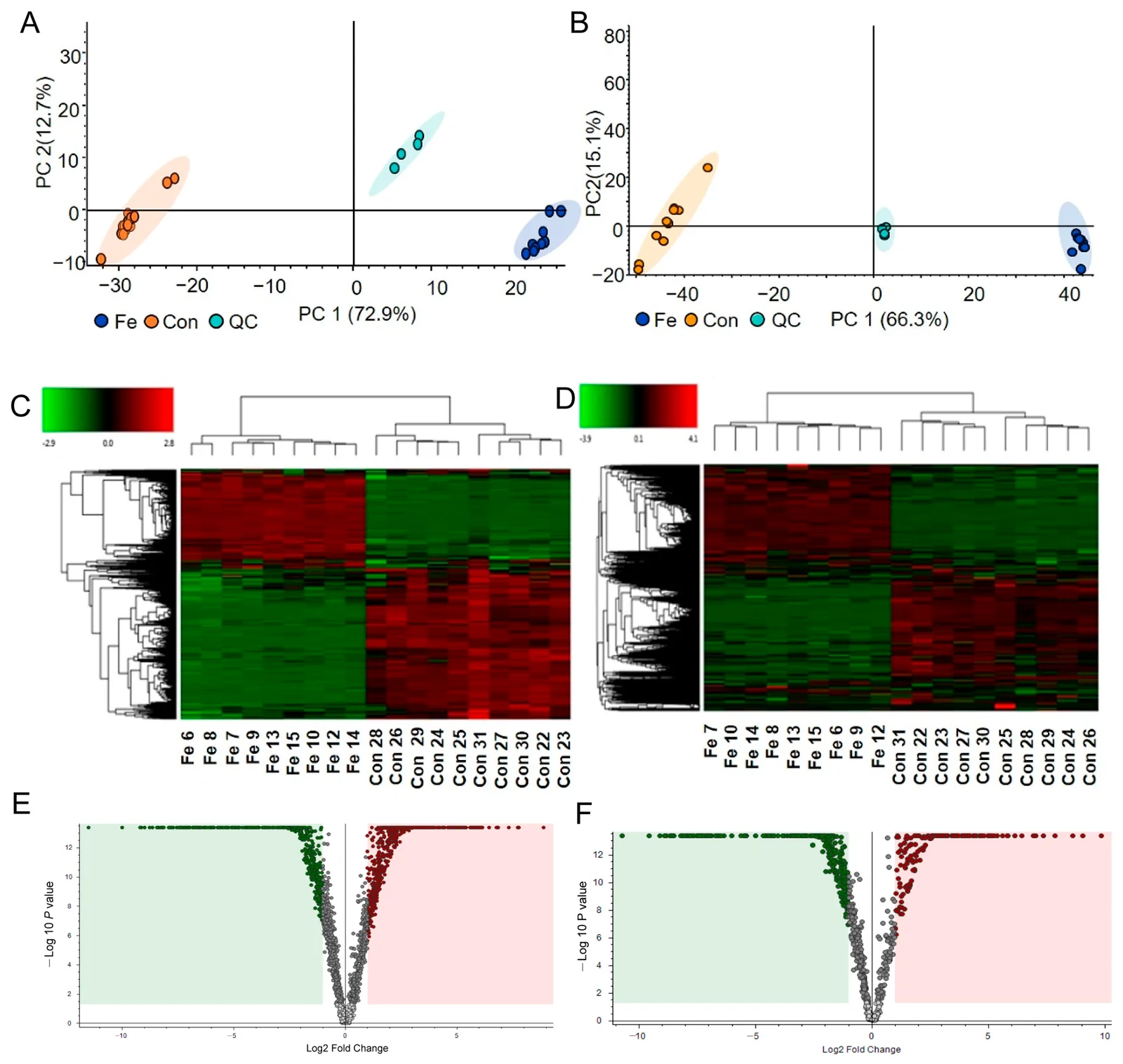
PCA and cluster analysis heat maps of control group and fermented ginseng feed group. (**A**) PCA in negative ion mode. (**B**) PCA in positive ion mode. (**C**) Cluster analysis heat map in negative ion mode. (**D**) Cluster analysis heat map in positive ion mode. Volcano plot comparing ginseng feed and fermented ginseng feed. (**E**) Positive ion mode; (**F**) Negative ion mode.

To elucidate the specificity of the fermented ginseng feed and its potential bioactive substance basis, a systematic comparison of the chemical profiles between the fermented ginseng feed and the conventional basal diet was conducted. Differential constituent analysis aimed to identify characteristic compounds that were either generated or significantly altered during fermentation, thereby providing a direct material basis for subsequent investigations into the mechanisms affecting broiler physiological functions. The total ion chromatograms are shown in Figure 3. As shown in Figure 4, fermented ginseng feed and basal feed were distinctly separated in both PCA and HCA, highlighting significant clustering effects. The PCA indicated clustering of QC samples, confirming the stability of the instrument throughout the analysis. A volcano plot was generated to identify compounds with significant differences; variables with a *p*-value < 0.05 and fold change > 3 were considered differentially expressed between the feeds. Multivariate statistical analysis identified 41 different compounds in fermented ginseng feed and basal feed. The differential compounds included amino acids, purines, long-chain fatty acid organic compounds, and panaxatriol (Table 2).

### 3.2. Growth Performance

To evaluate the effects of fermented ginseng feed on the growth performance of roosters, specifically, we determined the average daily gain, average daily feed intake, and feed conversion ratio to verify its potential as a functional feed additive for promoting growth and improving breed efficiency. The Fe group had significantly higher final body weight and average daily gain compared to the Con group (*p* < 0.05). The Fe group also showed a lower feed conversion ratio (*p* < 0.05), indicating better feed efficiency. In addition, the survival rate was significantly increased in the Fe group compared with the control group (*p* < 0.05) (Table 3).

### 3.3. Carcass Performance

Carcass performance is a critical indicator for evaluating economic returns and nutrient deposition efficiency in poultry production. Table 4 summarizes the carcass performance. The chest muscle rate and leg muscle rate of the Fe group were significantly different from those of the Con group (*p* < 0.05). Dressing percentage, semi-eviscerated percentage and eviscerated percentage in the Fe group both increased. However, these increases were not significant (*p* > 0.05).

### 3.4. Meat Quality

#### 3.4.1. Conventional Meat Quality

To identify the specific impacts of fermented ginseng by-product on the muscle quality of Hongyu roosters, the conventional meat quality traits of the breast muscle were further evaluated. As shown in Table 5, dietary supplementation with fermented ginseng by-product significantly increased water-holding capacity in the chest muscle quality of Hongyu roosters (*p* < 0.05). Compared with the Con group, shear force and drip loss were significantly reduced in the Fe group (*p* < 0.05). Table 6 shows the effect of fermented ginseng by-product feed on the chest muscle quality of Hongyu roosters. Compared with the Con group, the crude protein in the Fe group increased significantly (*p* < 0.05).

#### 3.4.2. Amino Acids

Amino acids are not merely essential precursors for protein synthesis and muscle accretion in poultry, but also function as critical regulators of metabolic pathways and immune responses. In Table 7, fermented ginseng by-product feed significantly altered the amino acid composition of breast muscle. Compared with the Con group, the Fe group exhibited higher levels of proline and valine (*p* < 0.05). Other amino acids did not change significantly (*p* > 0.05).

#### 3.4.3. Fatty Acid

Table 8 reveals the effect of feed with fermented ginseng by-products on fatty acid content in breast muscle of Hongyu roosters. Fermented ginseng feed did not significantly improve the levels of C16:0, C16:1, C18:0 and C18:1. However, the content of C18:2 with nutritional and health care value in the chest muscle increased significantly (*p* < 0.05), while C20:4 content was significantly lower (*p* < 0.05).

#### 3.4.4. Determination of Chest Muscle Fiber

**Figure 5:**
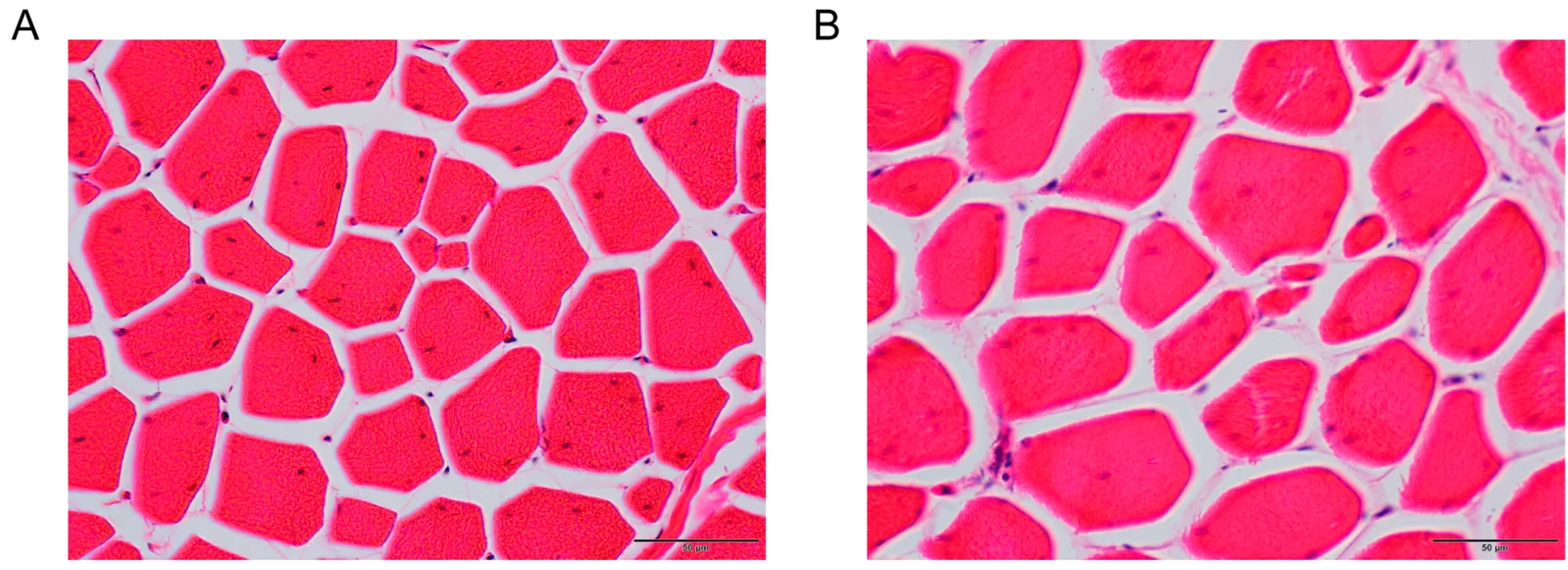
HE-stained chest muscle tissue sections. (**A**) Con group chest muscle tissue. (**B**) Fe group chest muscle tissue.

Histological analysis was performed to evaluate the effects of fermented ginseng by-product supplementation on the structural characteristics of pectoral muscle fibers. Compared with the Con group, the Fe group exhibited a significantly increased muscle fiber diameter (*p* < 0.05), whereas the muscle fiber density was significantly decreased (*p* < 0.05) (Table 9). Consistently, HE staining analysis revealed larger muscle fiber profiles in the Fe group compared with the Con group (Figure 5A). In contrast, the number of muscle fibers per unit area was reduced in the Fe group, as indicated by the lower muscle fiber density (Figure 5B).

### 3.5. Organ Index Determination

**Figure 6:**
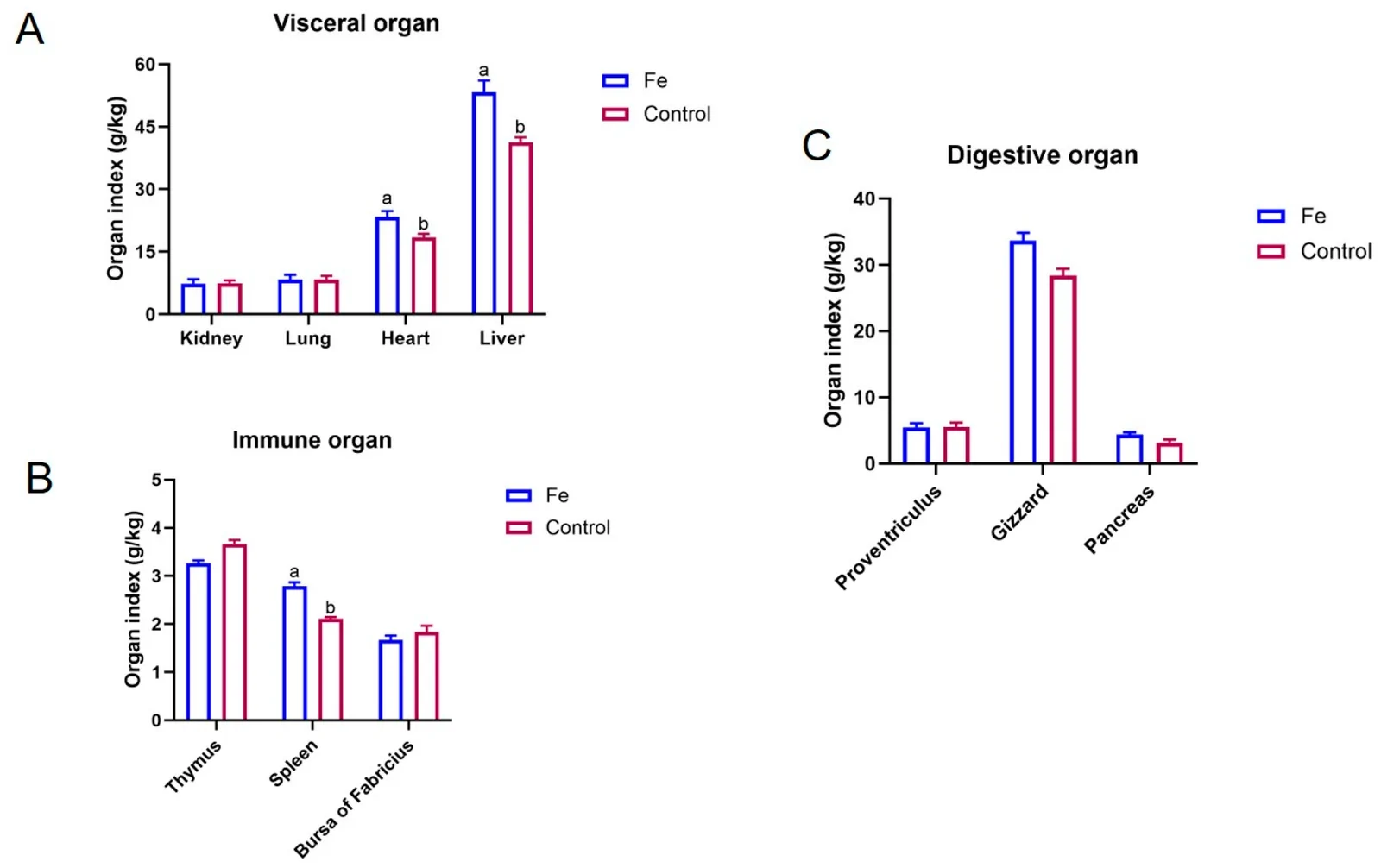
Effects of fermented ginseng by-product supplementation on organ indices of Hongyu roosters. (**A**) Visceral organ index. (**B**) Immune organ index. (**C**) Digestive organ index. ^a,b^ Different superscript letters indicate significant differences between groups (*p* < 0.05).

In order to comprehensively evaluate the effect of fermented ginseng feed on the physiological health of roosters and explore whether it can exert its biological activity by regulating organ development, we measured the organ indices of related visceral organs, digestive organs and immune organs. In Figure 6A, the liver and heart weight indices in the Fe group were significantly higher than those in the Con group (*p* < 0.05). The immune organs appeared normal, and the spleen index was significantly higher, as illustrated in Figure 6B. Digestive organs showed no noticeable abnormalities, as presented in Figure 6C.

### 3.6. Effect of Fermented Ginseng Feed on Blood Biochemical Indexes

To evaluate the nutritional metabolism and overall health status of roosters fed fermented ginseng feed, a comprehensive serum biochemical analysis was conducted. Hematological parameters in Table S1 show significant differences between the Fe and Con groups, with higher values for hemoglobin (%), erythrocyte sedimentation rate (ESR, mm/h), platelets (1000/mm^3^), leukocytes (1000/mm^3^), neutrophils (%), eosinophils (%), basophils (%), lymphocytes (%), monocytes (%), and total protein levels in the Fe group (*p* < 0.05).

### 3.7. Analysis for Sense Organ in Chest Muscle

**Figure 7:**
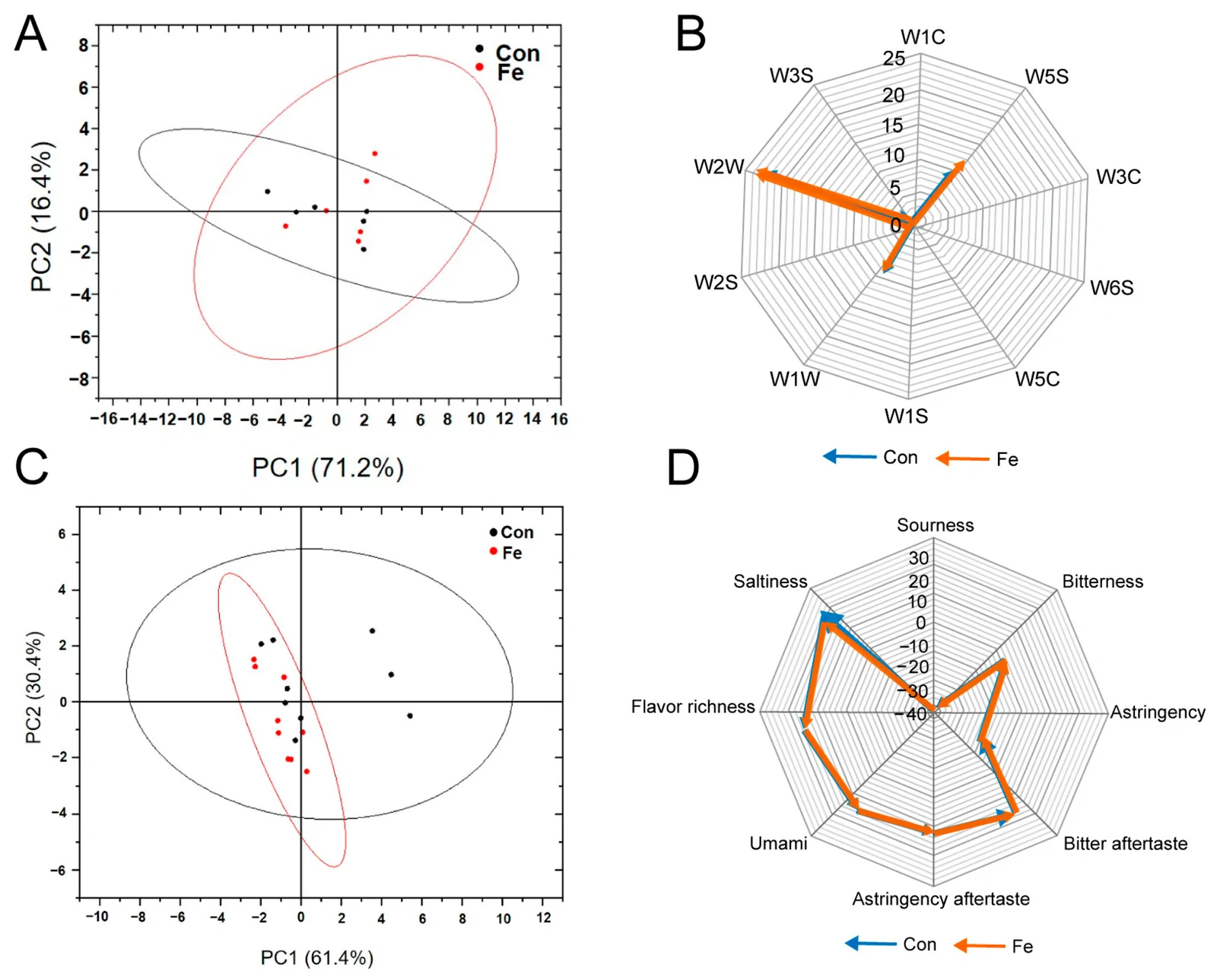
Differences in odor and taste profiles of chest muscle between Con and Fe groups. (**A**) PCA score plot of odor profiles. (**B**) Radar chart of electronic nose odor response values. (**C**) PCA score plot of taste profiles. (**D**) Radar chart of electronic tongue taste response values.

Feed palatability is a critical factor influencing feed intake and, consequently, the growth performance of roosters. The sensory profile of feed, characterized by its taste and aroma, directly determines its acceptance by animals. Therefore, we evaluated the sensory organs in the chest muscle. PCA results revealed a distinct separation between the Fe and Con groups based on aroma profiles, with sensors W1W, W2S, and W5S exhibiting strong responses to volatile compounds in the samples, suggesting high levels of sulfides, alcohols, aromatic compounds, and nitrogen oxides in the chicken breast (Figure 7A,B). The electronic tongue system, composed of five selective artificial lipid membrane sensors (CA0, C00, AE1, AAE, and CT0), was used to analyze sourness, bitterness and its aftertaste, astringency and its aftertaste, umami, and saltiness. PCA score chart of the electronic tongue (Figure 7C) and the taste radar chart (Figure 7D), based on taste response values (Table S2), revealed distinct differences in the taste profiles. Compared to the Con group, the Fe group had significantly lower bitterness and saltiness in chicken breast (*p* < 0.05), with a reduction in the bitterness aftertaste value as well. Regarding the tasteless point, defined as a taste index value of 0 below which a taste is undetectable, the results indicated that acidity, astringency, and aftertaste values fell below this threshold, making them unsuitable as effective evaluation indices for distinguishing between the two groups. Thus, these taste attributes did not significantly contribute to the sensory evaluation of the chicken samples (Table S3).

### 3.8. Serum Untargeted Metabolomics Analysis

**Figure 8:**
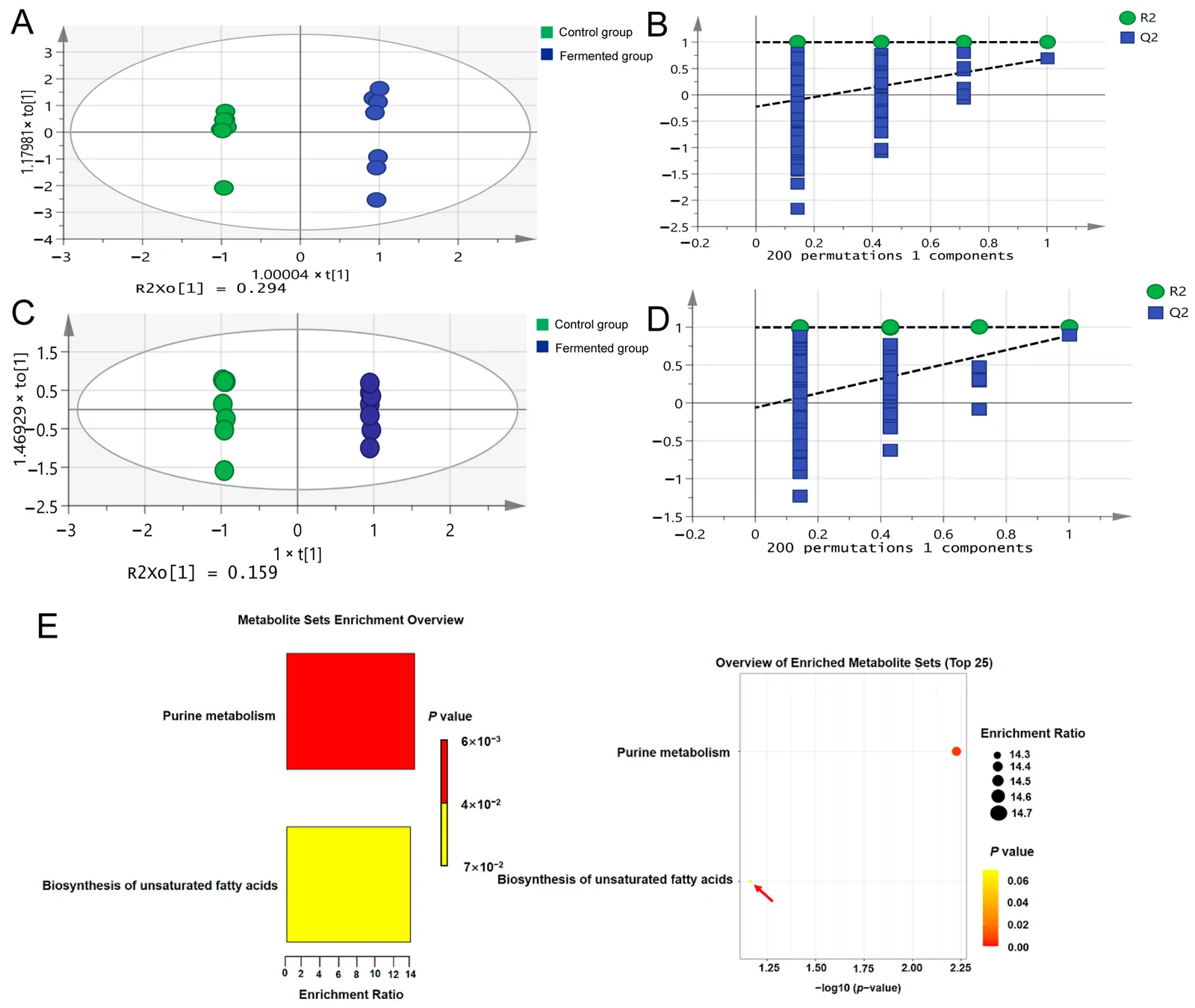
OPLS-DA analysis and KEGG metabolic pathway enrichment of blood samples from broilers (**A**) Negative ion mode. (**B**) A total of 200 permutation tests for negative ion mode. (**C**) Positive ion mode. (**D**) A total of 200 permutation tests for positive ion mode. (**E**) KEGG pathway enrichment.

To investigate the serum metabolite profile following the dietary supplementation of fermented ginseng by-products, we performed untargeted metabolomics on serum samples. Figure 8A–D showed a clear separation between the Fe and Con groups in OPLS-DA. The reliability of the OPLS-DA model was further evaluated by 200 permutation tests. The obtained R^2^Y and Q^2^ values indicated good model fitting and predictive performance, and the permutation results demonstrated that the model was not overfitted. A total of 15 differential metabolites were identified in the blood of roosters fed fermented ginseng feed. These metabolites primarily included phospholipids, amino acids, fatty acids, and phenylpropionic acid derivatives (Table 10). Pathway enrichment analysis of differential metabolites in the blood revealed pathways related to unsaturated fatty acid biosynthesis and purine metabolism (Figure 8E).

## 4. Discussion

In recent years, the development of sustainable alternative feed resources has received increasing attention due to the rising demand for animal production and concerns regarding environmental sustainability. Various alternative feed resources, including microalgae, aquatic plants, and insect-derived ingredients, have been investigated as potential substitutes for conventional feed ingredients. Microalgae supplementation has been reported to influence nutrient digestibility, rumen fermentation characteristics, blood metabolites, milk production, and fatty acid profiles in dairy cows [42]. Similarly, Azolla pinnata has attracted interest as a sustainable protein source owing to its favorable nutritional characteristics and potential to improve animal performance [43]. In addition, insect-based feed resources have been explored due to their high nutrient availability and potential contribution to circular agriculture [44]. These studies highlight the importance of identifying innovative and sustainable feed resources to improve livestock productivity while reducing environmental burdens [45].

Among these sustainable feed resources, the utilization of agricultural and medicinal plant by-products represents an attractive strategy for converting low-value residues into functional feed ingredients while promoting resource recycling and reducing environmental impacts. In the present study, dietary supplementation with fermented ginseng by-products significantly improved growth performance, enhanced feed efficiency, and positively influenced meat quality characteristics in Hongyu roosters. Metabolomic analysis further revealed that fermented ginseng by-product supplementation induced coordinated metabolic alterations, particularly in amino acid metabolism, lipid remodeling, and purine-related pathways. These findings indicate that fermented ginseng by-products may serve as a promising functional feed ingredient by improving nutrient utilization and regulating metabolic homeostasis in poultry. Although the experimental diets showed similar overall nutritional profiles, they were not strictly isoenergetic and isonitrogenous, and slight differences in nutrient composition may represent a potential limitation of the present study.

Metabolomic analysis further revealed that fermented ginseng by-product supplementation induced coordinated metabolic alterations, particularly in amino acid metabolism, lipid remodeling, and purine-related pathways. Beyond the improvements in growth performance and meat quality, metabolomic analysis provided further insights into the potential metabolic basis underlying the effects of fermented ginseng by-product supplementation. A total of 41 differential metabolites were identified, which were mainly associated with amino acid metabolism, lipid remodeling, nucleotide metabolism, and plant-derived bioactive compounds. The increased abundance of amino acid-related metabolites, including DL-citrulline, L-tyrosine, and 4-hydroxyisoleucine, may indicate enhanced amino acid availability and nitrogen metabolism, thereby potentially supporting muscle development and nutrient utilization. In addition, alterations in lipid-related metabolites, such as glycerol 3-phosphate and phytosphingosine, suggest possible regulation of lipid remodeling and membrane-associated metabolic processes [46]. Changes in purine-related metabolites, including guanosine and guanine, further indicate a potential adjustment of nucleotide metabolism and metabolic homeostasis. Moreover, increased levels of tryptophan-derived metabolites, including indole-3-acetic acid and DL-3-indolelactic acid, may reflect alterations in host–microbial metabolic interactions, although this possibility requires further validation. In conclusion, metabolomic analysis revealed that fermented ginseng by-product supplementation was associated with alterations in metabolic profiles, particularly pathways related to unsaturated fatty acid biosynthesis and purine metabolism. These findings suggest that fermented ginseng by-products have potential as a functional feed ingredient for improving animal production performance and provide insights into the metabolic responses associated with their supplementation. The improved FCR observed in Hongyu roosters suggests enhanced feed utilization efficiency following fermented ginseng by-product supplementation. Previous studies have indicated that fermented feed ingredients may improve nutrient availability through modulation of intestinal function and microbial activity. The enhanced feed efficiency observed in this study may potentially be associated with improved nutrient utilization and metabolic regulation induced by bioactive components derived from fermented ginseng by-products. But, because intestinal morphology, gut microbiota composition, and nutrient digestibility were not directly evaluated, these mechanisms remain speculative and require further validation. Secondly, previous studies have indicated that fermented Chinese herbal medicine by-products contain various bioactive metabolites, including organic acids and microbial-derived compounds, which may exert beneficial effects on intestinal health and nutrient utilization [47]. However, the effects of fermented feed supplementation on poultry performance are not always consistent. Previous studies have reported that fermented feed supplementation did not significantly improve growth performance in some poultry models, although beneficial effects on antioxidant status, immune function, digestive enzyme activity, or intestinal microbiota were observed [48]. The inconsistent responses may be attributed to differences in fermentation substrates, microbial strains, supplementation levels, fermentation conditions, bird breeds, and feeding periods. For example, different inclusion levels of fermented feed have been shown to produce variable effects on broiler growth performance, indicating that the dosage and composition of fermented products are critical factors influencing their nutritional efficacy [49].

Meat quality brings key economic benefits to the poultry industry [50]. In meat production systems, carcass characteristics are an important factor in evaluating production efficiency [51]. Interestingly, the fermented ginseng by-product group showed improved water-holding capacity, as indicated by the reduced drip loss. The increased muscle fiber diameter and altered muscle microstructure may contribute to changes in water distribution within muscle tissue. However, WHC is a complex trait influenced by multiple factors, including muscle pH, myofibrillar protein characteristics, structural integrity, and postmortem biochemical changes. Therefore, the improved WHC observed in the present study may result from the combined effects of these factors, and further investigations are required to elucidate the underlying mechanisms. Evidence from previous research indicates that increased muscle fiber diameter may be associated with higher shear force values in poultry meat, which may partly explain the increased shear force observed in the Fe group [52]. However, meat tenderness is a complex quality trait influenced by various factors, including connective tissue characteristics, collagen content and cross-linking, intramuscular fat deposition, sarcomere length, and postmortem proteolysis. Since these parameters were not measured in the present study, the contribution of muscle fiber enlargement to reduced tenderness should be interpreted with caution. Further studies are needed to clarify the mechanisms underlying the changes in meat texture induced by fermented ginseng by-product supplementation.

FAAs in muscle serve as pivotal flavor precursors and are intimately linked to muscle protein metabolism [53]. The Fe group has significantly elevated concentrations of proline and valine in breast muscle. This enrichment suggests an effect on both the potential palatability and the structural characteristics of the meat. Proline is characterized by a sweet and umami taste and accounts for 25% of collagen, which plays an important role in growth and wound repair [54]. Valine, a branched-chain amino acid (BCAA), has been reported to participate in the regulation of muscle protein metabolism and may influence anabolic processes through nutrient-sensing pathways, including the mTOR pathway [55]. Therefore, the increased valine concentration observed in the Fe group may reflect enhanced availability of amino acid substrates associated with muscle growth and protein turnover, which could partly relate to the increased muscle fiber diameter observed in histological analysis. However, the involvement of mTOR signaling requires further validation through measurements of pathway-related proteins and their phosphorylation status. Intriguingly, the increased levels of these amino acids may provide a metabolic basis for the improved muscle quality traits observed in the present study. Branched-chain amino acids, including valine, are recognized as important regulators of muscle protein metabolism and have been implicated in the regulation of anabolic processes through nutrient-sensing pathways. Thus, the increased valine abundance observed after fermented by-product supplementation may represent a potential contributor to enhanced muscle development, although the underlying signaling pathways require further investigation [55]. The increased abundance of these amino acids may provide a metabolic basis for the enhanced muscle quality traits observed in this study. Among them, proline is closely involved in extracellular matrix metabolism and collagen synthesis, suggesting that altered amino acid availability may influence muscle structural properties.

Dietary supplementation with fermented ginseng by-products significantly altered the fatty acid composition of Hongyu rooster meat. Specifically, the proportion of C18:2 (linoleic acid) was significantly increased in the Fe group compared with the control group (*p* < 0.05). C18:2 is an essential omega-6 polyunsaturated fatty acid that cannot be synthesized de novo in animals and must be obtained from dietary sources. It serves as an important component of cell membrane phospholipids and acts as a precursor for the biosynthesis of bioactive lipid mediators. The increased deposition of C18:2 in poultry meat suggests that fermented ginseng by-product supplementation may improve the nutritional value of chicken products by enhancing the accumulation of essential fatty acids. In contrast, the proportion of C20:4 (arachidonic acid) was significantly decreased in the Fe group compared with the control group (*p* < 0.05). C20:4 is a biologically active omega-6 polyunsaturated fatty acid derived from C18:2 through elongation and desaturation processes. Although arachidonic acid plays essential roles in maintaining cell membrane structure and physiological functions, excessive accumulation may promote the production of proinflammatory eicosanoids. Therefore, the reduction in C20:4 observed in the Fe group may indicate modulation of polyunsaturated fatty acid metabolic pathways and a shift in fatty acid composition toward a more balanced lipid profile. Collectively, these findings suggest that fermented ginseng by-product supplementation can regulate fatty acid metabolism and improve the overall nutritional profile of poultry products.

Serum biochemical indices reflect the metabolic status and systemic physiological condition of poultry. In this study, dietary supplementation with fermented ginseng by-products significantly increased RBC, hemoglobin, and hematocrit levels in Hongyu roosters. These improvements may be associated with the bioactive components derived from ginseng, as ginsenosides have been reported to participate in the regulation of hematopoietic processes. Moreover, fermentation may enhance the transformation and availability of certain ginseng-derived compounds, which could potentially contribute to the improved physiological status in poultry [56]. Concurrently, activation of the immune system was observed, evidenced by the marked elevation of white blood cells; lymphocytes; monocytes; and various granulocytes, including neutrophils, eosinophils, and basophils. Ginseng polysaccharides and probiotic metabolites are powerful immunomodulators that can trigger the proliferation and differentiation of immune cells [57]. The elevation of leukocyte profiles indicates that ginseng by-products enhanced the immunity of Hongyu roosters, thereby bolstering their resistance against environmental pathogens. Regarding serum protein metabolism, the significant increases in total protein, albumin, and α-globulin reflect an accelerated hepatic protein synthesis. Fermentation breaks down macromolecular proteins in ginseng by-products into easily absorbable small peptides and free amino acids, upgrading the nutritional value of the diet. The elevated serum albumin level serves as a reliable indicator of superior nutritional status and vigorous protein deposition, which strongly aligns with the increased breast and leg muscle yields observed in this study. Furthermore, the higher α-globulin levels and increased spleen index suggest an enhanced immune response. Moreover, electronic tongue analysis revealed that fermented ginseng by-product supplementation significantly altered the taste profile of chicken breast, characterized by reduced bitterness and bitterness aftertaste compared with the control group. This improvement may be related to fermentation-induced alterations in bitter compounds from ginseng by-products, as ginsenosides, amino acids, peptides, and proteins are considered major contributors to ginseng bitterness [58].

To further elucidate the metabolic mechanisms underlying the beneficial effects of fermented ginseng by-product on Hongyu roosters, untargeted serum metabolomics was performed. Differential metabolites were mainly enriched in purine metabolism and the biosynthesis of unsaturated fatty acids, indicating that dietary supplementation primarily affected nucleotide and lipid metabolic homeostasis. The coordinated reduction in adenine, uric acid, and 5-hydroxyisouric acid suggests a suppression of purine catabolism, which may reflect improved energy homeostasis and reduced oxidative stress. In parallel, the increased abundance of phospholipid metabolites, including lecithin, sphingomyelin, and phosphatidylcholine, together with alterations in fatty acid-related metabolites, indicates membrane lipid remodeling and enhanced cellular membrane stability. In addition, the altered levels of hippuric acid and 3-phenyllactic acid may indicate that fermented ginseng by-product supplementation is associated with changes in host–microbial metabolic interactions. Collectively, these metabolic alterations suggest that fermented ginseng by-product may contribute to improved metabolic homeostasis through the regulation of nucleotide metabolism and lipid remodeling, while potential involvement of microbiota-related mechanisms requires further investigation.

Although fermented ginseng by-products showed promise as a functional feed ingredient for improving growth performance and metabolic characteristics, several practical considerations must be addressed before commercial application. The availability and consistency of ginseng by-product resources may vary depending on ginseng processing scale and production regions. In addition, the economic feasibility of fermentation processes, including substrate preparation, microbial culture, processing costs, and quality control, requires further evaluation. Large-scale production and application studies are also needed to determine whether the beneficial effects observed under experimental conditions can be maintained in commercial farming systems. Therefore, future studies integrating cost–benefit analysis, standardized fermentation protocols, and large-scale feeding trials will be essential to promote the practical utilization of fermented ginseng by-products as sustainable feed resources.

## 5. Limitations

Several limitations of the present study should be acknowledged. First, only one inclusion level (3%) of fermented ginseng by-product was evaluated; therefore, the optimal supplementation dose and dose–response relationship require further investigation. Second, this study was conducted using only male Hongyu roosters, and whether similar effects occur in female birds or other poultry breeds remains unclear. The lack of monitoring of fermentation parameters, such as pH variation during fermentation, represents a limitation of the present study. Meanwhile, body weight was measured only at the beginning and the end of the experimental period, which limited our ability to evaluate the dynamic changes in growth performance during different stages of fermented ginseng by-product supplementation. More frequent measurements, such as weekly body weight monitoring, would provide a more detailed understanding of growth trajectories and temporal responses to dietary supplementation. Third, although metabolomic analysis provided insights into the metabolic alterations associated with fermented ginseng by-product supplementation, the relatively limited sample size warrants further validation using larger cohorts. In addition, intestinal microbiota composition and related functional changes were not evaluated, limiting our understanding of the potential gut-mediated mechanisms. Furthermore, a formal a priori power analysis based on expected effect sizes and variability was not conducted before the experiment, which represents another limitation of the present study. Although five replicate pens per treatment were used as independent experimental units, the statistical power associated with this experimental design was not quantitatively determined. Future studies incorporating power-based experimental designs and additional independent replicates may further strengthen the validation of these findings. Another limitation of this study is that the experimental diets were not strictly isoenergetic and isonitrogenous, as the inclusion of fermented ginseng by-product residue slightly altered the nutrient composition of the diets. Therefore, some effects observed in growth performance and metabolic profiles may have been partially influenced by differences in dietary nutrient levels rather than solely by the bioactive properties of the fermented product. Future studies using diets precisely matched for energy and nitrogen content are warranted to further validate the specific effects of fermented ginseng by-product supplementation. Finally, several proposed biological mechanisms, including pathways related to muscle development and nutrient metabolism, require further confirmation through molecular analyses such as gene expression and protein-level validation. Future studies incorporating these approaches will provide a more comprehensive understanding of the nutritional and functional value of fermented ginseng by-products in poultry production.

## 6. Conclusions

In summary, this study highlights the potential of dietary supplementation with fermented ginseng by-products as a sustainable approach for improving meat quality and nutritional value in Hongyu roosters. Microbial fermentation modified the chemical composition of ginseng by-products, resulting in the identification of 41 differential chemical components. The fermented diet significantly improved growth performance, feed conversion efficiency, and meat quality in Hongyu roosters. Serum metabolomic analysis revealed alterations in metabolic profiles associated with fermented ginseng by-product supplementation, particularly pathways related to purine metabolism and unsaturated fatty acid biosynthesis. These findings provide a solid foundation for transforming traditional Chinese medicine by-products into poultry feed, thereby contributing to the development of high-quality and sustainable animal production.

## Figures and Tables

**Table 1:** Composition and nutrient level of experimental diet.

Ingredient Composition (%)	Con	Fe
Corn	61.9	59.4
Calcium bicarbonate	1.3	1.3
Dregs of bean	30	30
Soya bean oil	3.5	3.5
Limestone	1.5	1.0
Salt	0.3	0.3
Premix	1.5	1.5
Fermented ginseng by-product	/	3
Total	100	100
Nutrient Level		
Metabolizable energy (MJ/kg)	12.71	12.54
Crude protein (%)	18.3	18.9
Calcium (%)	0.89	0.88
Total phosphorus (%)	0.52	0.51
Lysine (%)	1.02	1.13
Methionine (%)	0.41	0.44

Note: Premix provides nutrition for each kilogram of diet: vitamin A, 9000 IU; vitamin D, 3500 IU; vitamin E, 35 IU; vitamin K, 32.2 mg; vitamin B12, 15 mg; vitamin B6, 50 mg; nicotinic acid, 35 mg; pantothenic acid, 10 mg; folic acid, 1 mg; thiamine, 3 mg; riboflavin, 5 mg; biotin, 0.5 mg; choline, 1000 mg; and vitamin C, 10 mg.

**Table 2:** Differential compounds in fermented *ginseng* feed and basal feed identified via UHPLC-HRMS.

No.	tR (min)	Molecular Weight	Formula	Ppm	Mode	Up/Down-Regulated	Identification
1	0.71	175.09483	C_6_H_13_N_3_O_3_	−4.91	[M − H]	up	DL-Citrulline
2	0.73	196.05762	C_6_H_12_O_7_	−3.50	[M − H]	down	D-Gluconic acid
3	0.83	129.07921	C_6_H_11_NO_2_	1.80	[M + H]	up	Pipecolic acid
4	0.99	172.01369	C_3_H_9_O_6_P	0.09	[M + H]	up	Glycerol 3-phosphate
5	0.99	293.11107	C_11_H_19_NO_8_	0.03	[M + H]	up	N-Acetylmuramic acid
6	1.01	250.13177	C_13_H_18_N_2_O_3_	0.10	[M + H]	down	N-Caffeoylputrescine
7	1.03	283.09158	C_10_H_13_N_5_O_5_	−0.30	[M − H]	down	Guanosine
8	1.03	175.08368	C_7_H_13_NO_4_	−4.46	[M − H]	up	Calystegine B2
9	1.11	151.04945	C_5_H_5_N_5_O	0.23	[M + H]	down	Guanine
10	1.12	161.06878	C_6_H_11_NO_4_	−0.18	[M + H]	up	α-Aminoadipic acid
11	1.19	146.03683	C_9_H_6_O_2_	0.35	[M + H]	up	Coumarin
12	1.19	178.06301	C_10_H_10_O_3_	0.09	[M + H]	up	(E)-4-Methoxycinnamic acid
13	1.20	136.05243	C_8_H_8_O_2_	−0.03	[M + H]	up	4-Methoxybenzaldehyde
14	1.28	147.08937	C_6_H_13_NO_3_	−1.16	[M + H]	up	4-Hydroxyisoleucine
15	1.45	198.05240	C_9_H_10_O_5_	−2.15	[M − H]	up	Vanillyl mandelic acid
16	1.61	181.07393	C_9_H_11_NO_3_	0.18	[M + H]	up	L-tyrosine
17	1.65	153.07897	C_8_H_11_NO_2_	−0.08	[M + H]	up	Octopamine
18	1.92	208.15753	C_12_H_20_N_2_O	−0.14	[M + H]	down	Ammodendrine
19	2.22	167.05827	C_8_H_9_ NO_3_	0.18	[M + H]	up	Pyridoxal
20	2.29	219.11039	C_9_H_17_NO_5_	−1.30	[M − H]	down	D-pantothenic acid
21	2.53	222.10025	C_11_H_14_N_2_O_3_	−0.88	[M − H]	up	Glycyl-L-phenylalanine
22	2.80	166.11055	C_9_H_14_N_2_O	−0.39	[M + H]	up	2-sec-Butyl-3-methoxypyrazine
23	3.93	280.10614	C_13_H_16_N_2_O_5_	0.78	[M + H]	down	L-α-Aspartyl-L-phenylalanine
24	4.96	166.06301	C_9_H_10_O_3_	0.09	[M + H]	up	Paeonol
25	6.35	162.08843	C_7_H_14_O_4_	−4.81	[M − H]	up	Monobutyrin
26	7.67	192.07871	C_11_H_12_O_3_	0.37	[M + H]	up	Myristicin
27	7.83	196.07363	C_10_H_12_O_4_	0.38	[M + H]	up	Ethyl Vanillate
28	8.95	175.06260	C_10_H_9_NO_2_	−4.17	[M − H]	up	Indole-3-acetic acid
29	9.08	162.03091	C_9_H_6_O_3_	−4.83	[M − H]	up	Umbelliferone
30	9.37	203.11522	C_9_H_17_NO_4_	−2.63	[M − H]	up	Acetylcarnitine
31	10.42	205.07399	C_11_H_11_NO_3_	0.49	[M + H]	up	DL-3-Indolelactic acid
32	11.91	161.08407	C_10_H_11_NO	0.06	[M + H]	up	Tryptophol
33	12.44	390.13163	C_20_H_22_O_8_	0.43	[M − H]	up	Abruquinone B
34	12.60	237.10021	C_12_H_15_NO_4_	0.42	[M + H]	up	Ethopabate
35	16.12	372.12094	C_20_H_20_O_7_	0.09	[M + H]	up	3,5,7,3,4′–Pentamethoxyflavone
36	16.91	354.11031	C_20_H_18_O_6_	−0.09	[M + H]	up	Albanin A
37	23.07	228.13603	C_12_H_20_O_4_	−0.58	[M + H]	up	Traumatic Acid
38	28.87	278.18771	C_17_H_26_O_3_	−1.76	[M + H]	up	Paradol
39	31.51	332.25622	C_18_H_36_O_5_	−0.15	[M − H]	down	Phloionolic acid
40	34.70	317.29259	C_18_H_39_NO_3_	−1.27	[M + H]	up	Phytosphingosine
41	37.86	476.38566	C_30_H_52_O_4_	−1.89	[M + H]	up	Panaxatriol

**Table 3:** Effect of growth performance.

Items	Con	Fe	*p*-Value
Initial Weight (g)	682.70 ± 0.45	684.50 ± 0.34	0.08
Final Weight (g)	2927 ± 5.65 ^b^	3354 ± 6.41 ^a^	0.035
Average Daily Gain (g)	22.45 ± 0.44	23.70 ± 0.38	0.246
Average Daily Feed Intake (g)	64.6 ± 0.28	70.3 ± 0.65	0.073
Feed Conversion Ratio	2.88 ± 0.31 ^a^	2.63 ± 0.23 ^b^	0.021
Survival Rate (%)	88 ± 1.98 ^b^	94 ± 2.06 ^a^	0.019

^a,b^ Different superscript letters within the same row indicate significant differences between groups (*p* < 0.05).

**Table 4:** Effect of fermented ginseng by-product feed on carcass performance of Hongyu roosters.

Items	Con	Fe	*p*-Value
Live body weight (g)	2602 ± 39.46	3046 ± 26.78	0.463
Dressing percentage (%)	87.78 ± 0.98	89.68 ± 1.58	0.068
Semi-eviscerated percentage (%)	81.05 ± 1.27	82.56 ± 0.92	0.052
Eviscerated percentage (%)	69.31 ± 1.57	70.09 ± 0.59	0.056
Chest muscle rate (%)	11.53 ± 0.90 ^b^	12.92 ± 0.41 ^a^	0.026
Leg muscle rate (%)	23.53 ± 1.03 ^b^	25.27 ± 0.78 ^a^	0.04
Abdominal fat rate (%)	3.21 ± 0.15	2.96 ± 0.24	0.067

^a,b^ Different superscript letters within the same row indicate significant differences between groups (*p* < 0.05).

**Table 5:** Effect of fermented ginseng by-product feed on chest muscle quality of Hongyu roosters.

Items	Con	Fe	*p*-Value
pH_45min_	5.81 ± 0.04	5.76 ± 0.07	0.070
pH_24h_	5.67 ± 0.06	5.63 ± 0.08	0.289
L_24 h_	50.40 ± 1.67	49.20 ± 1.10	0.063
a_24 h_	9.60 ± 2.07	10.60 ± 2.30	0.552
b_24 h_	10.00 ± 1.00	9.60 ± 1.14	0.074
Shear force (N)	33.34 ± 2.04 ^b^	36.63 ± 1.14 ^a^	0.025
Water-holding capacity (%)	40.23 ± 1.25 ^b^	42.01 ± 0.73 ^a^	0.048
Drip loss (%)	3.07 ± 0.16 ^a^	2.60 ± 0.39 ^b^	0.039

^a,b^ Different superscript letters within the same row indicate significant differences between groups (*p* < 0.05).

**Table 6:** Effects of fermented ginseng by-product supplementation on the proximate composition of breast muscle.

Items (%)	Con	Fe	*p*-Value
Moisture	73.13 ± 0.24	72.71 ± 0.46	0.69
Crude protein	21.90 ± 0.40 ^b^	22.75 ± 0.47 ^a^	0.04
Crude fat	2.52 ± 0.15	2.37 ± 0.06	0.241
Ash	1.28 ± 0.02	1.31 ± 0.05	0.116

^a,b^ Different superscript letters within the same row indicate significant differences between groups (*p* < 0.05).

**Table 7:** Differences in breast muscle amino acid content of broilers between the Con and Fe groups (g/100 g).

Items	Con	Fe	*p*-Value
Aspartic acid	2.20 ± 0.10	2.25 ± 0.17	0.152
Threonine	1.03 ± 0.05	1.08 ± 0.05	0.095
Serine	0.84 ± 0.04	0.88 ± 0.04	0.223
Glutamic acid	3.78 ± 0.17	3.93 ± 0.26	0.149
Proline	0.71 ± 0.01 ^b^	0.80 ± 0.01 ^a^	0.021
Glycine	0.98 ± 0.06	1.10 ± 0.08	0.336
Alanine	1.23 ± 0.07	1.23 ± 0.09	0.167
Valine	1.19 ± 0.07 ^b^	1.28 ± 0.07 ^a^	0.025
Methionine	0.53 ± 0.03	0.57 ± 0.02	0.068
Isoleucine	1.13 ± 0.06	1.20 ± 0.06	0.502
Leucine	1.81 ± 0.09	1.91 ± 0.10	0.495
Tyrosine	0.80 ± 0.04	0.83 ± 0.03	0.287
Phenylalanine	1.06 ± 0.07	1.14 ± 0.06	0.488
Histidine	1.50 ± 0.07	1.52 ± 0.12	0.379
Lysine	1.54 ± 0.08	1.47 ± 0.11	0.128
Arginine	1.46 ± 0.08	1.57 ± 0.07	0.420
Total amino acids	21.79 ± 0.94	22.75 ± 1.27	0.771

^a,b^ Different superscript letters within the same row indicate significant differences between groups (*p* < 0.05).

**Table 8:** Effect on fatty acid content in chest muscle.

Component	Con	Fe	*p*-Value
C16:0 (%)	25.43 ± 2.85	26.60 ± 0.62	0.144
C16:1 (%)	3.10 ± 0.55	3.64 ± 0.45	0.291
C18:0 (%)	12.47 ± 1.96	9.10 ± 1.64	0.547
C18:1 (%)	33.23 ± 2.62	36.43 ± 1.91	0.713
C18:2 (%)	15.73 ± 2.01 ^b^	20.33 ± 1.91 ^a^	0.035
C20:4 (%)	10.01 ± 2.38 ^a^	3.86 ± 2.10 ^b^	0.028

^a,b^ Different superscript letters within the same row indicate significant differences between groups (*p* < 0.05).

**Table 9:** Effect of fermented ginseng feed on chest muscle fiber.

Items	Con	Fe	*p*-Value
Muscle fiber diameter (μm)	35.70 ± 0.45 ^b^	39.57 ± 0.32 ^a^	0.033
Muscle fiber density (root/mm^2^)	671.09 ± 20.38 ^a^	609.24 ± 25.67 ^b^	0.026

^a,b^ Different superscript letters within the same row indicate significant differences between groups (*p* < 0.05).

**Table 10:** Differential compounds in serum metabonomics identified by UHPLC/Q-Orbitrap MS.

No.	tR (min)	Molecular Weight	Formula	Ppm	Mode	Up/Down	Function	Identification
1	0.89	168.02776	C_5_H_4_N_4_O_3_	−3.45	[M − H]	down	End products of purine metabolism	Uric acid
2	0.91	184.02280	C_5_H_4_N_4_O_4_	−2.45	[M − H]	down	Purine derivative	5-hydroxyisouric acid
3	4.53	260.13762	C_11_H_20_N_2_O_5_	1.54	[M + H]	down	—	L-gamma-Glutamyl-L-leucine
4	9.03	166.06245	C_9_H_10_O_3_	−3.26	[M − H]	down	—	3-Phenyllactic acid
5	9.71	179.05847	C_9_H_9_NO_3_	1.26	[M + H]	down	Considered as a biomarker of aging	Hippuric acid
6	14.06	430.30870	C_27_H_42_O_4_	0.91	[M + H]	down	Anti-inflammatory, antifungal and gastric protective effects	Hecogenin
7	16.09	178.06310	C_10_H_10_O_3_	0.60	[M + H]	down	Liver protection, antidiabetic, anticancer, neuroprotective, antibacterial, antioxidant, etc.	4-Methoxycinnamic acid
8	16.21	757.56203	C_42_H_80_NO_8_ P	−0.17	[M + H]	up	Commonly used in the adjuvant treatment of fatty liver, atherosclerosis and other diseases; anti-aging and improving brain function	Lecithin
9	16.27	730.59884	C_41_H_83_N_2_O_6_P	−0.05	[M + H]	up	Regulation of transmembrane signal transduction; inhibition of phospholipase Cδ1	N-stearoylsphingomyelin
10	16.36	733.56235	C_40_H_80_NO_8_P	0.27	[M + H]	up	—	DL-Dipalmitoylphosphatidylcholine
11	16.46	135.05464	C_5_H_5_N_5_	1.10	[M + H]	down	—	Adenine
12	16.88	228.13614	C_12_H_20_O_4_	−0.10	[M − H]	up	Reduce lipid accumulation in human adipocytes	Traumatic Acid
13	17.26	334.28762	C_22_H_38_O_2_	1.31	[M − H]	down	Have potential benefits for human health	Docosatrienoic acid
14	17.93	284.27189	C_18_ H_36_ O_2_	1.28	[M − H]	down	—	Stearic acid
15	19.21	339.35030	C_22_H_45_NO	0.53	[M + H]	down	—	Docosanamide

## Data Availability

The original contributions presented in this study are included in the article/Supplementary Materials. Further inquiries can be directed to the corresponding authors.

## Supplementary Materials

The following supporting information can be downloaded at: https://www.mdpi.com/article/10.3390/vetsci13080844/s1, Table S1: Determination of blood biochemical indexes. Table S2: Performance of PEN3 electronic nose sensor and determination index; Table S3: Characteristics and performance of SA-402B electronic tongue sensor.

## Abbreviations

The following abbreviations are used in this manuscript:

AGC	Automatic gain control.
AKT	Protein Kinase B.
HCA	Hierarchical cluster analysis.
HE	Hematoxylin–eosin.
HESI	Heated electrospray lonization.
HMDB	Human metabolome database.
KEGG	Kyoto Encyclopedia of Genes and Genomes.
OPLS-DA	Orthogonal partial least squares discriminant analysis.
PCA	Principal component analysis.
UHPLC-HRMS	Ultra-high performance liquid chromatography–high-resolution mass spectrometry.
WHC	Water-holding capacity.
FAAs	Free amino acids.
