# Supplementary material for: Effects of Fermented Ginseng By-Product Supplementation on Growth Performance, Meat Quality, Flavor, and Fatty Acid Metabolism in Hongyu Roosters
Source: Vet Sci. 2026 Aug 21;13(8):844. doi: 10.3390/vetsci13080844 (PMC13517448; doi:10.3390/vetsci13080844)
Supplement: Supplementary file 1 [file vetsci-13-00844-s001.zip › vetsci-4453054-supplementary.pdf]

Supplementary Material

Effects of Fermented Ginseng By-product Supplementation on Growth Performance, Meat Quality, Flavor, and Fatty Acid Metabolism in Hongyu Roosters

Hongxi Chen [1,†], Yuhao Liu [2,†], Shuo Zhang [1,†], Yi Jin [2], Wanfeng Liang [2], Xijiu Jin [2,*], Jun Zhe Min[1,*]

[1] Key Laboratory of Natural Medicines of the Changbai Mountain, Ministry of Education, Department of Phamaceutical Analysis, College of Pharmacy, Yanbian University, Yanji 133002, Jilin Province, China

[2] Department of Animal Science, College of Agriculture, Yanbian University, Yanji 133002, Jilin Province, China

***** Correspondence: junzhemin23@163.com; jinxj@ybu.edu.cn

[†] These authors contributed equally to this work and should be regarded as co-first authors.

Table S1. Determination of blood biochemical indexes.

Items	Con	Fe	p-Value
Red Blood Cell (10^6/mm^3)	3.25	3.544	0.175
Hemoglobin (%)	46.2^b	79.4^a	0.038
Hematocrit	28.2^b	52^a	0.044
Erythrocyte sedimentation (mm/h)	0.522^b	0.658^a	0.029
Blood platelet (10^3/mm^3)	53^b	72.8^a	0.048
White blood cell (10^3/mm^3)	24.6^b	47.62^a	0.024
Neutrophil (%)	26.6^b	52.8^a	0.01
Eosinophilic granulocyte (%)	4.38^b	6.16^a	0.047
Basophils (%)	2.61^b	4.48^a	0.01
Lymphocyte (%)	56.02^b	64.36^a	0.027
Monocyte (%)	1.8^b	4.44^a	0.005
Total protein (g/L)	58.18^b	70.54^a	0.049
Albumin (g/L)	28.3^b	52.36^a	0.018
α-globulin (g/L)	0.246^b	0.538^a	0.024
β-globulin (g/L)	0.437	0.473	0.357
γ-globulin (g/L)	0.1475	0.1173	0.128
Na (mmol/L)	150.6	142.2	0.249
K (mmol/L)	5.1	5.2	0.268
Cl (mmol/L)	129.4	128	0.653
Inorganic phosphorus (mmol/L)	2.304	2.418	0.246
Ca (mmol/L)	3.76^b	4.48^a	0.037
Mg(mmol/L)	0.938^b	1.292^a	0.048
Glucose(mmol/L)	9.098	9.398	0.295
Urea nitrogen(mmol/L)	3.146^b	3.682^a	0.034

Note: Values in the same row without or with the same letter superscripts indicate no significant difference ($p > 0.05$). Values with different small letter superscripts indicate a significant difference ($p < 0.05$).

Table S2. Performance of PEN3 electronic nose sensor and determination index.

Sensor	Con	Fe	*p*-Value	Detection component
W1C	0.63 ± 0.01	0.63 ± 0.01	0.148	Aromatic components, benzene
W5S	12.56 ± 0.25^{b}	14.10 ± 1.51^{a}	0.027	Extremely sensitive to nitrogen oxides
W3C	0.19 ± 0.01	0.19 ± 0.01	0.241	Sensitive aromatic components, ammonia
W6S	0.16 ± 0.01	0.15 ± 0.01	0.315	Mainly selective for hydrides.
W5C	0.27 ± 0.01	0.27 ± 0.01	0.194	Aromatic components of short-chain alkanes
W1S	0.27 ± 0.02^{b}	0.31 ± 0.03^{a}	0.048	Sensitive to methyl groups
W1W	7.16 ± 0.03^{B}	7.35 ± 0.07^{A}	0.022	Sensitive to sulfide
W2S	0.27 ± 0.02^{B}	0.32 ± 0.03^{A}	0.039	Sensitive to alcohols, aldehydes and ketones
W2W	23.42 ± 0.12^{b}	23.92 ± 0.40^{a}	0.047	Aromatic component, sensitive to organic sulfide
W3S	1.59 ± 0.03^{A}	1.54 ± 0.01^{B}	0.033	Sensitive to long-chain alkanes

Note: Values in the same row without or with the same letter superscripts indicate no significant difference ($p > 0.05$). Values with different small letter superscripts indicate a significant difference ($p < 0.05$).

Table S3. Characteristics and performance of SA-402B electronic tongue sensor.

Items	Con	Fe	p-Value
Sour	-32.05 ± 2.55	-30.50 ± 0.70	0.108
Bitter	0.55 ± 0.16[a]	0.32 ± 0.22[b]	0.033
Puckery	-22.11 ± 1.09	-21.00 ± 0.22	0.295
Bitter aftertaste	7.41 ± 2.28	5.87 ± 0.59	0.177
Puckery aftertaste	0.08 ± 0.17	-0.05 ± 0.17	0.069
Delicate	3.69 ± 0.22	3.62 ± 0.19	0.187
Freshness richness	12.68 ± 0.87	12.62 ± 0.56	0.081
Salt	25. 11 ± 2.72[a]	23.04 ± 0.53[b]	0.044

Note: Values in the same row without or with the same letter superscripts indicate no significant difference ($p > 0.05$). Values with different small letter superscripts indicate a significant difference ($p < 0.05$).
